# Effect of High Pressure Microfluidization on the Crystallization Behavior of Palm Stearin — Palm Olein Blends

**DOI:** 10.3390/molecules19045348

**Published:** 2014-04-24

**Authors:** Lijuan Han, Lin Li, Bing Li, Lei Zhao, Guoqin Liu, Xinqi Liu, Xuede Wang

**Affiliations:** 1 College of Light Industry and Food Sciences, South China University of Technology, Guangzhou 510640, Guangdong, China; E-Mails: hanlijuan.scut@gmail.com (L.H.); felinli@scut.edu.cn (L.L.); bli@scut.edu.cn (B.L.); liuxinqi2014@163.com (X.L.); 2 College of Food Sciences, South China Agricultural University, Guangzhou 510642, Guangdong, China; E-Mail: scauzl@scau.edu.cn; 3 College of Food Science and Technology, Henan University of Technology, Zhengzhou 450001, Henan, China; E-Mail: wangxuede1962@126.com

**Keywords:** high-pressure microfluidization, palm stearin, palm olein, crystallization behavior

## Abstract

Moderate and high microfluidization pressures (60 and 120 MPa) and different treatment times (once and twice) were used to investigate the effect of high-pressure microfluidization (HPM) treatment on the crystallization behavior and physical properties of binary mixtures of palm stearin (PS) and palm olein (PO). The polarized light microscopy (PLM), texture analyzer, X-ray diffraction (XRD) and differential scanning calorimetry (DSC) techniques were applied to analyze the changes in crystal network structure, hardness, polymorphism and thermal property of the control and treated blends. PLM results showed that HPM caused significant reductions in maximum crystal diameter in all treated blends, and thus led to changes in the crystal network structure, and finally caused higher hardness in than the control blends. The XRD study demonstrated that HPM altered crystalline polymorphism. The HPM-treated blends showed a predominance of the more stable β' form, which is of more interest for food applications, while the control blend had more α- and β-form. This result was further confirmed by DSC observations. These changes in crystallization behavior indicated that HPM treatment was more likely to modify the crystallization processes and nucleation mechanisms.

## 1. Introduction

High-pressure microfluidization (HPM) is an emerging technology, which makes use of a device called a microfluidizer. This device uses a high-pressure positive displacement pump (the pressure vrange is approximately 5–200 MPa) [1]. This equipment has been traditionally used in the pharmaceutical industry to make pharmaceutical emulsions, as well as in the food industry to produce nanosystems [2,3] or homogenized proteins (milk, whey protein, trypsin, *etc.*) [4,5,6,7] and dietary fiber [8] only in the last few years. High-pressure microfluidization uses the combined forces of high-velocity impact, high-frequency vibration, instantaneous pressure drop, intense shear, cavitation, and ultra-high pressures up to 200 MPa with a short treatment time (less than 5 s) and continuous operation [9,10]. Therefore, it differs from high hydrostatic pressure (HHP), which only uses ultra-high pressures from 100 to 1500 MPa [11], but has some vibration and cavitation similarities with sonication. Oh *et al.* [12] found that HHP treatments of cocoa butter crystallized in Form V (one of the polymorphic forms of triacylglyceride crystals in cocoa butter) crystals with 100, 300, or 600 MPa pressure did not alter the rate of Form V to Form VI transitions of cocoa butter. However, information on the effects of HMP on lipid crystallization behavior is not available.

Lipid crystallization behavior has very important implications, mainly for industrial processing of products whose physical properties depend largely on the presence of fat crystals, such as chocolates, margarines, and shortenings. The crystallization process is divided into nucleation and crystal growth phases. Nucleation involves the formation of molecule clusters that exceed a critical size and are therefore stable. Fats’ tendency to crystallize is of fundamental concern to processing techniques. Triacylglycerols (TAG) generally crystallize initially into the α and β' polymorphic forms, although the β form is more stable. The polymorphic transformation is an irreversible process from the less stable to the more stable form, and depends on the temperature and time involved [13]. Fats with crystals in the β' form offer greater functionality, because they are softer, support aeration better, and offer creaming properties. Thus, generally β' form is the polymorph of greatest interest for producing high-fat foods. However, as long as proper processing methods are adopted, suitable products can be obtained even using fats with a high propensity towards the β form [14]. Higaki *et al.* used ultrasonic irradiation to compare the crystallization behavior of tripalmitoylglycerol and cocoa butter before and after the treatment, and put forward the notion that sonication affects the crystal nucleation processes [15]. Since HPM has similar vibration and caviation effects as sonication, we dared to speculate that like sonication, HPM would affect the crystal nucleation processes of lipids through the following mechanisms: (i) violent collapse of impact bubbles may form active sites for nucleation; (ii) cooling caused by evaporation from the surface of the cavity during the growth of a cavitation bubble may increase supercooling; (iii) local pressure may increase the melting point in the vicinity of a collapsing cavity, which means that the degree of supercooling is increased; (iv) tiny crystals resulting from intense shear can also form active sites for nucleation [15]. It is clear that retarding polymorphic transformation in solid fats can help, at least for some time, to delay loss in quality [16]. Therefore, if HPM can retard polymorphic transformation of lipid from β' form to β form by altering the crystal nucleation processes, and a suitable product with greater functionality will be obtained. Once a crystalline nucleus has formed, it begins to grow by incorporating other molecules [13]. 

Since crystallization behavior was often related to the important aspect of the physical properties of oils and fats [17], therefore, if the crystal size, crystal morphology, crystal polymorphism and crystal network structure of the system are changed, it would eventually affect the performance of the final product. The aim of this study was thus to gain more insight into the effect of HPM on the crystallization behavior, microstructure, and macroscopic properties of binary PS/PO blends. Therefore, a series of HPM treatment conditions (*i.e*., varying treatment pressure and treatment time) were analyzed with several techniques to assess their effects on the TAG crystallization behavior and texture during storage at 0 °C for varied lengths of time (*i.e*., 0 h, 4 h, 1 day and 5 days). Polarized light microscopy (PLM) provided information on the development of TAG crystal microstructure, while X-ray diffraction was applied to detect the polymorphic forms during the crystallization process. Texture was evaluated in terms of hardness determined by penetrometry. Differential scanning calorimetry (DSC) was applied to investigate the melting behaviors of the control and the treated samples.

## 2. Results and Discussion

### 2.1. Polarized Light Microscopy (PLM)

**Figure 1:**
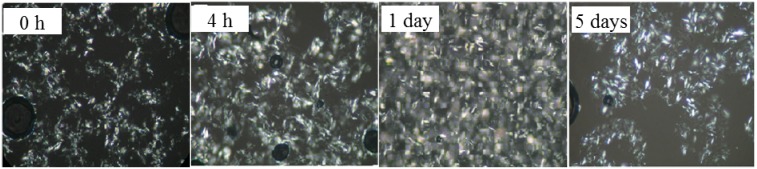
Greyscale PLM images of PS/PO oil control blend crystal networks stored for various periods: 0 h (onset of storage), 4 h, 1 day, 5 days. Magnification 500×.

**Figure 2:**
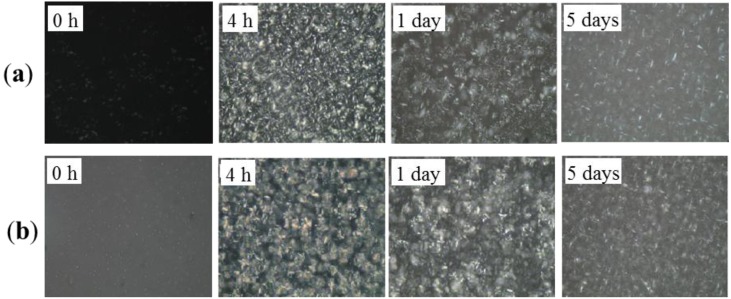
Polarized light photomicrographs for the PS/PO oil blends under 60 MPa HPM treatment: (**a**) Treated once and crystallized 0 h, 4 h, 1 day and 5 days. (**b**) Treated twice and crystallized 0 h, 4 h, 1 day and 5 days respectively. Magnification 500×.

**Figure 3:**
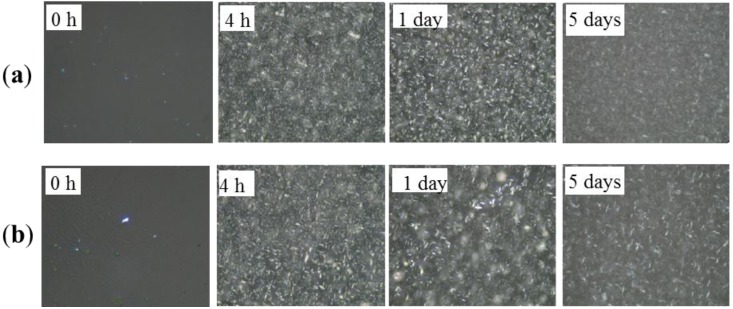
Polarized light photomicrographs for the PS/PO oil blends under 120 MPa HPM treatment: (**a**) Treated once and crystallized 0 h, 4 h, 1 day and 5 days. (**b**) Treated twice and crystallized 0 h, 4 h, 1 day and 5 days respectively. Magnification 500×.

PLM was used to examine the morphology of the crystallized systems. Crystal size distributions were observed in the control blends and the processed blends as a function of storage time using polarized light microscopy with a magnification of 500×. The PLM recorded of the control sample and those prepared under two pressures (60 MPa and 120 MPa) stored for varied length of time (0 h, 4 h, 1 day and 5 days) ae shown in Figure 1, Figure 2 and Figure 3, respectively. The control blends were full of spherocrystal particles, and aggregated clusters after a period of storage (5 days). Different significantly from the control samples, crystal particle size was small and needle-like in the treated samples. The microfluidization produced a much more rigid microstructure in comparison with that of the control sample, which stabilized the β' form. Figure 2 and Figure 3 show that crystal size decreased with the treatment pressure in the pressure range investigated; we argue that this decrease might result from a size decrease in the nuclei with treatment pressure. As we have said above, these smaller nuclei were associated with tiny bubbles due to high-pressure impact. The smaller the nuclei the more incomplete crystal growth is in the same storage time. This would lead to a less compact 3D crystal network structure than the one developed under lower pressure.

### 2.2. Changes in Polymorphism

**Figure 4:**
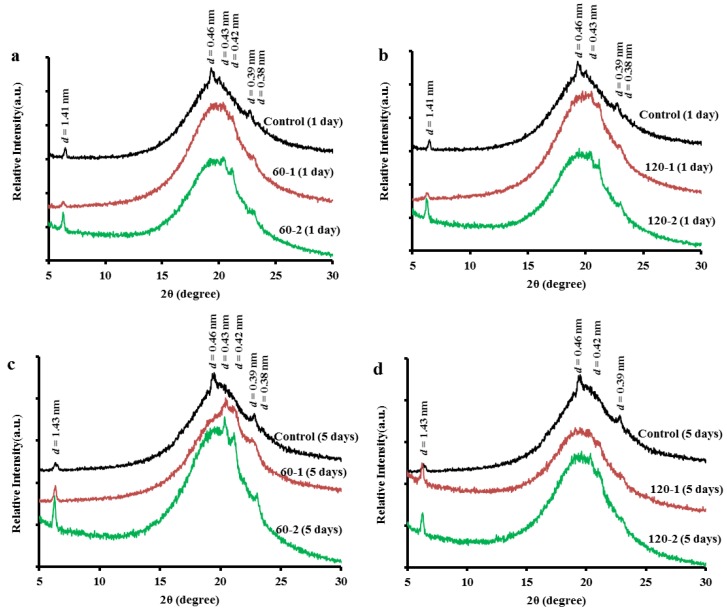
X-ray diffraction patters for PS/PO samples crystallized at 0 °C for 1 day (**a**, **b**) and 5 days (**c**, **d**) treated under 60 MPa (**a**, **c**) and 120 MPa (**b**, **d**), respectively (all the curves were shifted for clarity).

X-ray diffraction is often used as a technique to detect \changes in polymorphism, helping outline applications for the fat bases produced. The α-form is characterized by one strong short spacing (*d* values marked in Figure 4) line in the XRD pattern near *d* = 0.42 nm. The β'-form is characterized by two strong short spacing lines at *d* = 0.37–0.40 nm and at *d* = 0.42–0.43 nm. The β-form is characterized by a strong lattice spacing line at near *d* = 0.46 nm and a number of other strong lines around *d* = 0.36–0.39 nm [18].

Diffractograms of the control blends and the treated blends crystallized for different times (1 day and 5 days) were shown in Figure 4a–d. For the control blends’ crystals (Figure 4, Control-1 day and Control-5 days), the XRD peak of β form (*d* = 0.46 nm) did not change for both crystallization times. Since the β form is more stable than the α and β' polymorphic forms, and the polymorphic transformation is an irreversible process from the less stable to the more stable form, that crystalline polymorph form in control blends will not change unless the storage temperature changes. The processed samples showed two peaks of varying intensity at *d* = 0.42–0.43 nm and 0.38 nm, which corresponded to the occurrence of the β' polymorph, probably mixed with α crystals. This phenomenon was most obvious in the samples treated at 60 MPa and stored for 5 days (*i.e*., 60-2 (5 days)). Oppositely, the XRD peak of *d* = 0.46 nm decreased in intensity or disappeared after HPM treatment. This indicated that these changes in crystallization behavior of TAG molecules were correlated with the HPM treatment. However, the small-angle XRD peaks of *d* = 1.40–1.43 nm did not change after the HPM treatment.

Figure 4 also showed that the control blends reached crystallization completion (indicated by the appearance of β polymorph) earlier than all the treated samples subjected to the same storage time. This might have a correlation with the large crystal particles that initially existed in these control blends (as shown in Figure 1, 0 h) forming the crystal nuclei. Based on the XRD results (Figure 4), and the analysis of the PLM images, the small crystals were associated with the β' polymorph (in the processed samples) and the large crystals or clusters were associated with the β polymorph (in the control samples). The findings of this study were in agreement with those reported by Narine *et al.* [19] that polymorphism is one of the important factors that influence the microstructure of fats. 

### 2.3. Hardness

**Figure 5:**
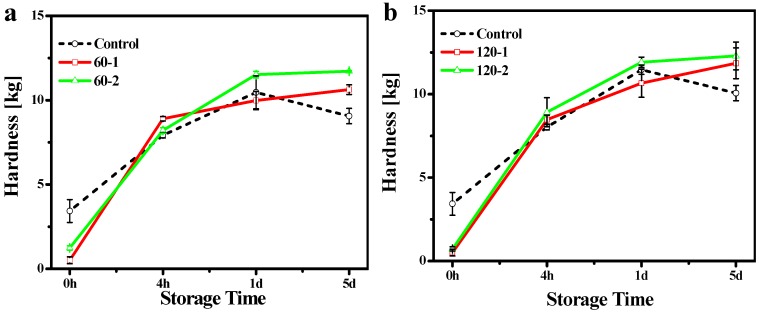
Hardness of control blend and different treated pressures (**a**: 60 MPa and **b**: 120 MPa) *vs.* treated times (1 and 2) as a function of storage time (0 h, 4 h, 1 day and 5 days) at 0 °C obtained with penetrometry. Values indicated the mean for two replicates.

In order to study the influence of the changes in the crystallization behavior discussed above on the physical properties of the blend system, penetration tests were used to determine the hardness of the samples. The hardness of a fat is an important property that strongly influences the perceived texture of the fat-containing food product [20]. Figure 5 showed the hardness of control blend and treated blends as a function of storage time after crystallization. The hardness was governed not only by the amount of solid fat present in the network, but also by the structure of this network [21]. Thus, we could explain the variation of the hardness through the microstructures of this network by PLM images (Figure 1, Figure 2 and Figure 3). After crystallization for 4 hr and 1 d, the control blend (Figure 1, 4 h and 1 day) and the 60 MPa series (Figure 2, 4 h and 1 day) had similar crystal network microstructures, thus the hardness of these samples were much closer. Since the fat would crystallize further during storage that it would lead to a denser crystal network (Figure 2, 5 days), and correspondingly lead to a higher hardness (*p* < 0.05) (see Figure 5a, 5 days). Different polymorphic forms often show different microstructures [22], so polymorphism could be an explanation for the change in hardness (see X-ray results): the β'-form crystal was detected in the 60 MPa series after crystallizaion for 5 days (Figure 4c), and generally, β'-form and small size conferred a fine crystal network [23]. This meant that for treated blends, the interactions between the fat phase and the solid dispersed phase create a firmer network. In contrast, β-form crystals were formed in the corresponding control blend (see Figure 4c) and gave rise to larger aggregates (see Figure 1, 5 days), and this could be the reason for the worse interaction with the solid dispersed phase and the reason for evolution of less condensed fat crystal network microstructures and a lower hardness (*p* < 0.05) during storage. The same hardness trends were observed in the 120 MPa series.

### 2.4. Changes in Melting Properties

**Figure 6:**
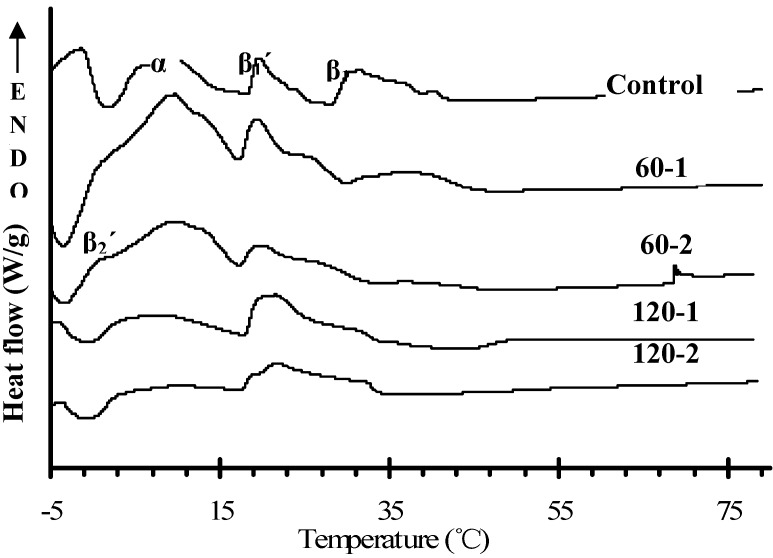
Dynamic heating thermograms (5 °C/min) for control blend and treated blends stored for 5 days.

All heating thermograms obtained by DSC for both control blend and treated blends stored for 5 days are shown in Figure 6. Because of the complexity of the thermogram, we refer here to the peak-top melting point. According to Che Man *et al.* [24], the high temperature endothermic peaks during the heating thermogram for PS/PO represent polymorphic states β_1_' and β_1_, while the low-temperature melting peaks represent polymorph states β_2_' and α. Based on their studies and our observations using the X-ray diffraction technique (see Section 2.3), we estimated the polymorphic forms of PS/PO blends for control and treated samples as shown in Figure 6. If the peak temperatures of control and treated blends heating thermograms were compared, it could be seen that after HPM treatment, the β_1_' forms was bigger and broader in the control blend than in treated blends, and the peak height of α form decreased with increasing treatment pressure. Figure 6 also agreed with the TAG compositions of PS and PO. Che Man *et al.* [24] studied the heating thermograms of triglyceride standards that POO showed two peaks of which the higher was at 15.05 °C and PPO showed a single peak at around 25 °C. On the basis of this result, the following melting peaks could be identified: two major endothermic peaks of the heating thermogram for PS/PO were assigned to POO (≈10 °C) and PPO (≈20 °C), respectively. The positions of these two peaks were found to shift to lower temperatures in our study. This behavior, as previously mentioned, was probably associated with melting of tiny crystals corresponding to intense shear generated by the HPM treatment.

## 3. Experimental

### 3.1. Materials

Refined bleached deodorized (RBD) palm stearin (PS) characterized by an iodine value (IV) of 8.00, melting point (MP) of 51.9 °C, acid value (AV) of 0.10 mg KOH/g, and RBD palm olein (PO, IV 48.30, MP 26.8 °C, AV 0.22 mg KOH/g) were provided by Kerry Grain and Oil Co. (Guangzhou, China). Both PS and PO were used without further purification. The TAG compositions of PS were, in decreasing order of concentration, PPO/POP (23.10%), POO (16.76%), PPP (12.46%), POSt/PStO (9.78%), PPM (9.32%), PLP (8.56%), LaLaO (7.50%) and DLaLa (4.46%); and the TAG compositions of PO were PPO/POP (29.39%), POO (27.72%), PPM (8.70%), LaLaO (7.17%), PLP (6.97%), POSt/PStO (6.83%) and DLaLa (5.23%) [17], where: D, capric acid; La, lauric acid; M, myristic acid; P, palmitic acid; St, stearic acid; O, oleic acid and L, linoleic acid. Palm stearin contained more trisaturated and less monosaturated TAG, while palm olein contained more disaturated and less trisaturated TAG.

### 3.2. Methods

#### 3.2.1. Sample Preparation

Palm oil is considered to be the most economical and abundant edible oil worldwide in the near future and has been widely used as cooking oil, margarine, shortening in cooking, confectionery, bakery, *etc.* [25]. Because palm stearin (PS) and palm olein (PO) were the most common form of palm oil fractions, they were chosen as the starting oil in this study. The blends of PS and PO, with 26% (wt/vol) PS content were processed under the following conditions: 100 mL PO was preheated at 60 °C for 30 min (ensuring no existence of crystals) and then cooled to 40 °C (the temperature that HPM could withstand), 26 g PS was blended into this system (in order to make the PS/PO blends flow at the HPM treatment temperature, the amount of PS could not be too much), and stirred for 10 min at 500 rpm. Then, the corresponding blend was subjected to HPM (M-110EH-30 Microfluidizer Processor, Newton, MA, USA) once or twice under two pressures of 60 MPa and 120 MPa, respectively. Since the interior of the microfluidizer was equipped with a cooling loop device, the obtained sample temperature was controlled to about 40 °C (±5 °C). After that, the control blend and the treated blends were all cooled to 0 °C at 5 °C/min and then stored at 0 °C for various periods: 0 hours (0 h), 4 hours (4 h), 1 day (1 day) and 5 days (5 days). For convenient expression, different HPM treatment conditions were abbreviated as followed: 60-1 and 60-2 represented PS/PO blend treated separately for once and twice under 60 MPa; 120-1 and 120-2 represented PS/PO blend treated separately for once and twice under 120 MPa.

#### 3.2.2. Polarized Light Microscopy

Polarized light microscopy (PLM) was used to examine the morphology of the crystallized systems. To guarantee a uniform sample thickness, two cover slips were glued to a glass microscope slide with a distance of 2.2 cm between them. The sample (40 °C) was placed within this gap of a preheated (40 °C) glass slide, and a glass cover slip was placed over the sample such that it rested on the glued cover slips. After 30 min at 40 °C, the system was cooled to 0 °C and stored for various periods. Photomicrographs as a function of storage time was obtained of the slide, with a polarized light microscope equipped with a color video camera (model Power Shot G5, Canon Inc., Tokyo, Japan). The pictures were taken at a magnification of 500×.

#### 3.2.3. Hardness (Penetration Test)

The hardness of crystallized samples (20 mL in aluminium case crystallized under different treated conditions and stored at 0 °C for different time) was determined with a penetration test on a Texture Analyzer TA.XT plus (Stable Micro Systems Ltd., Surrey, UK) with a cylindrical probe (diameter = 6 mm). The probe penetrated the product at a constant speed of 2 mm/s to a distance of 6 mm. Hardness was defined as the maximum penetration force (kg), and each measurement was executed two times.

#### 3.2.4. X-ray Diffraction

The crystal polymorphic form of the fat sample was determined according to AOCS method Cj 2-95 [26]. The analyses were performed on a Phillips diffractometer (D8 ADVANCE, Bruker AXS Inc., Karlsruhe, Germany), using Bragg-Bretano geometry (θ:2θ) with Cu-Kα radiation (λ = 1.54056 Å, at 40 kV and 40 mA). Measurements were taken at 0.02° step size at 2θ and 17.7-s acquisition time with 3 to 30° scans (2θ scale). Polymorphic forms were identified from characteristic crystal short spacing (*d*). The α form displayed a single diffraction line at 0.415 nm. The β' form is characterized by two strong diffraction lines at 0.38 and 0.42 nm, while the β form is associated with a series of diffraction lines, one prominent at 0.46 nm and lines of lesser intensity at 0.37 and 0.38 nm [26,27,28]. Peak detection and analysis were obtained using the MDI Jade 5.0 software without baseline subtraction.

#### 3.2.5. Determination of Melting Curves

Melting behaviors were investigated using a TA Q100 differential scanning calorimeter (TA Instruments, New Castle, DE, USA). The calibration of the instrument was done by indium and zinc. Purge nitrogen (99.99%) was the carrier gas at a flow rate of 40 mL/min. The control and treated PS/PO blend samples (26% wt/vol) were all stored at 0 °C for 5 days. A sample of 3 to 5 mg was weighed into aluminum pan with lid and covered. An empty pan was used as a reference. The sample was then heated from −5 °C to 80 °C at a scan rate of 5 °C/min.

## 4. Conclusions

In this study, the crystallization behavior (crystal size, crystal polymorphism, *etc.*) and physical properties of binary PS/PO blends before and after HPM treatment was determined and analyzed. HPM treatment could increase the hardness of PS/PO blends, and this increase was a little more evident by increasing treatment time. However, there was little difference between the hardness obtained under moderate and high microfluidization pressures. The initial size of the crystal nuclei was less than the size of the untreated sample due to the high pressure treatment, and this thus retarded the overall crystallization rate. Treatment under high microfluidization pressure (*i.e*., 120 MPa) could not form an efficient crystal network compared with moderate pressure. After enough storage and crystal growth, the 60 MPa series exhibited a pronounced β' form, while in the 120 MPa series, although there was no significant amount of β' form, it had not attained the characteristic β form. 

The results indicated that HPM treatment could be used as an additional processing method to modify the physicochemical properties of PS/PO blends, such as microstructure, texture, and polymorphic forms. This new technology has the potential to be used in the production of healthier lipid sources, such as no *trans*- and low-saturated fat to tailor their functional properties to specific applications.
